# Do electronic health records affect the patient-psychiatrist relationship? A before & after study of psychiatric outpatients

**DOI:** 10.1186/1471-244X-10-3

**Published:** 2010-01-08

**Authors:** Randall F Stewart, Philip J Kroth, Mark Schuyler, Robert Bailey

**Affiliations:** 1 Health Sciences Library & Informatics Center, MSC09 5100, 1 University of New Mexico, Albuquerque, New Mexico 87131-0001, USA; 2 Department of Psychiatry, MSC09 5030e, 1 University of New Mexico, Albuquerque, NM 87131, USA; 3 Department of Internal Medicine, MSC10 5550, 1 University of New Mexico, Albuquerque, NM 87131, USA

## Abstract

**Background:**

A growing body of literature shows that patients accept the use of computers in clinical care. Nonetheless, studies have shown that computers unequivocally change both verbal and non-verbal communication style and increase patients' concerns about the privacy of their records. We found no studies which evaluated the use of Electronic Health Records (EHRs) specifically on psychiatric patient satisfaction, nor any that took place exclusively in a psychiatric treatment setting. Due to the special reliance on communication for psychiatric diagnosis and evaluation, and the emphasis on confidentiality of psychiatric records, the results of previous studies may not apply equally to psychiatric patients.

**Method:**

We examined the association between EHR use and changes to the patient-psychiatrist relationship. A patient satisfaction survey was administered to psychiatric patient volunteers prior to and following implementation of an EHR. All subjects were adult outpatients with chronic mental illness.

**Results:**

Survey responses were grouped into categories of "Overall," "Technical," "Interpersonal," "Communication & Education,," "Time," "Confidentiality," "Anxiety," and "Computer Use." Multiple, unpaired, two-tailed t-tests comparing pre- and post-implementation groups showed no significant differences (at the 0.05 level) to any questionnaire category for all subjects combined or when subjects were stratified by primary diagnosis category.

**Conclusions:**

While many barriers to the adoption of electronic health records do exist, concerns about disruption to the patient-psychiatrist relationship need not be a prominent focus. Attention to communication style, interpersonal manner, and computer proficiency may help maintain the quality of the patient-psychiatrist relationship following EHR implementation.

## Background

The current emphasis on the adoption and use of Electronic Health Records (EHRs) is well known. The Institute of Medicine advocated for EHR use as early as 2001 [1]. The Bush administration created the Office of the National Coordinator for Health Information Technology and set the goal of nationwide EHR implementation by 2014 [2,3]. The American Recovery and Reinvestment Act of 2009 will provide $20 billion in funding for health information technology, while at the same time stipulating that physician practices which do not use a certified EHR by 2014 may forfeit up to 3% of their Medicare reimbursements [4]. Recent Medicare and Medicaid legislation provides a 2% incentive for physicians to implement e-prescribing by 2009, while instituting a 2% penalty for those that do not by 2012 [5].

In spite of the improving costs of initial investment, barriers to EHR adoption remain [6]. Among these are effects on eye contact, time with the patient, and clinical workflow [7,8]; lack of interoperability between different EHR systems [9,10]; the need for training and the effects on time utilization [11]; culture changes, changes in the distribution of power, and user resistance [12]; uncertain or equivocal benefits [13,14]; and the introduction of new errors and other types of unintended consequences [15,16].

Patient satisfaction, however, does not seem to be a barrier. Since the 1980s, numerous studies have shown little change to overall patient satisfaction when physicians use computers in a clinical setting [17-23]. Patients generally seem to accept the use of computers in the delivery of their care. Some more recent studies have indicated an increase in patient satisfaction when EHRs are used [24,25]. Other studies have shown, however, that certain aspects of the patient-physician relationship are altered by computer use. Communication style becomes less fluent [26-29] and concerns about confidentiality of the health record increase [22,30-34]. Some early studies suggested that computer use may lead to increases (or smaller decreases) in anxiety over the course of an outpatient encounter [35-37] or that physicians who use computers during encounters are seen as "less ideal" than those who don't [38].

Unfortunately, psychiatric patients may be disproportionately influenced by these changes. The patient-psychiatrist relationship is arguably more reliant on communication skills, confidentiality, and psychodynamic interpretations than non-psychiatric specialties. Makoul [39] found that electronic records may lead to more "complete" documentation, but that there was a non-significant decrease in the amount of "patient-centered" communication and exploration of psychosocial issues. Changes to communication pattern [40] or eye contact [41] could conceivable lead practitioners to overlook or misinterpret the verbal and non-verbal cues which often lead to refined lines of inquiry. Similarly, physical placement of computer equipment (such as in corners, or around the perimeter of a room) could make sustained observation of patient behavior difficult, or lead to changes in the psychiatrist's body language that patients might misinterpreted as disinterest. The stigma against mental illness may magnify patients' concerns about confidentiality, leading to less open or less truthful communication [33,40,42]. This could subsequently alter screening for suicide or other high-risk events. Because symptoms of anxiety are associated with diagnoses of depression, bipolar disorder, schizophrenia, substance use, and posttraumatic stress disorder, changes in anxiety, brought about by EHR use, could potentially alter the accurate evaluation of these disorders. The "idealism" study by Cruickshank [38], performed in the United Kingdom in the early 1980s, is of uncertain significance today. It could represent discomfort with the emerging technology of the desktop computer, or the desire for a more traditional approach to medicine. More recent studies, however, have likened the computer to a "third party" in the examination room, altering the physicians' focus on the patient and altering the quality of the therapeutic dyad [43-45].

We found no studies which looked exclusively at the effect of EHR use on the relationship between the patient and his or her psychiatrist. This study investigates the effect of EHR use among psychiatric outpatients. A group of 161 psychiatric outpatients completed satisfaction surveys prior to EHR adoption and another 141 completed surveys at least 4 months following EHR adoption. The primary objective was to examine the correlation between EHR use and aspects of the patient-psychiatric relationship. We hypothesized that EHR use would decrease patient satisfaction scores related to communication, confidentiality, and anxiety.

## Methods

### Study Design

We used a quasi-experimental, pre-test and post-test design approved by the University of New Mexico (UNM) Health Sciences Center Human Research Review Committee (HRRC No. 04-365). The quasi-independent variable was exposure to paper charting (before an EHR implementation) or electronic charting (after implementation). The dependent variable was the quality of the patient-psychiatrist relationship as measured by a self-administered, paper-based questionnaire. Patient primary diagnosis was also recorded as a covariate.

### Instrument & Data Collection

Because of its ease of administration and its public availability, we chose the Rand Corporation's previously validated Patient Satisfaction Questionnaire-18 (PSQ-18) as a starting point in survey design [46]. The PSQ-18 captures seven dimensions of satisfaction, including "General Satisfaction," "Technical Quality," "Interpersonal Manner," "Communication," "Financial Aspects," "Time Spent with Doctor," and "Accessibility and Convenience." In order to control for acquiescence bias, the PSQ-18 applies balanced keying, in which both positively and negatively worded questions are included. Subjects record their responses on a five-point Likert scale ranging from "Strongly Agree" (1) to "Strongly Disagree" (5). During scoring, the scores for positively-worded questions are reversed so that for all questions, low scores consistently indicate low satisfaction and high scores consistently indicate high satisfaction.

We included all of the original PSQ-18 questions except for those in the "Financial Aspects" and "Accessibility & Convenience" subscales. We removed those questions since the literature review did not suggest that EHR use would change patients' attitudes towards these factors. Where necessary to make questions psychiatric specific, we replaced the word "medical" with "psychiatric." "Doctor" or "physician" was likewise replaced with "psychiatrist." This resulted in a draft of only 12 questions. Next, we added questions from an unpublished and unvalidated survey which had been locally drafted during study inception. This locally drafted survey included all of the PSQ-18 subscales as well as three additional subscales of "Anxiety," "Computer use," and "Confidentiality." The resulting composite draft, consisting of both PSQ-18 and locally drafted questions, included 49 questions.

Because questions on the locally drafted survey had been rationally derived without statistical analysis, we solicited feedback on survey design and understanding from a convenience sample of six inpatient volunteers from the UNM Psychiatric Center inpatient wards. We used the feedback to re-word confusing questions and to rank the questions by importance as perceived by the patients. In the final survey, we included all of the PSQ-18 questions (except for those in the "Financial Aspects" and "Accessibility & Convenience" subscales), and retained only enough of the highest-ranking local questions in order to yield a one-page survey that included at least two questions in each subscale. This final, composite survey contained 23 questions, 12 from the PSQ-18 and 11 from the local survey. The questions and subscales of the final survey are shown in Table 1. We retained the original PSQ-18 Likert scale and practice of balanced keying.

**Table 1:** Survey subscales and questions

Subscales & questions	Original PSQ-18 subscale*
*Overall*:	
The psychiatric care I have been receiving is just about perfect.	General satisfaction
I am dissatisfied with some things about the psychiatric care I receive.	General satisfaction

*Technical*:	
I have some doubts about the ability of the psychiatrists who treat me.	Technical quality
Sometimes psychiatrists make me wonder if their diagnosis is correct.	Technical quality
My psychiatrist could be a lot better.	local
I think my psychiatrist's office has everything needed to provide complete psychiatric care.	Technical quality
When I go for psychiatric care, they are careful to check everything when treating and examining me.	Technical quality

*Interpersonal*:	
Psychiatrists act too businesslike and impersonal toward me.	Interpersonal manner
I wish that I had a different psychiatrist.	local
My psychiatrist treats me in a very friendly and courteous manner.	Interpersonal manner

*Communication & Education*:	
Psychiatrists sometimes ignore what I tell them.	Communication
My psychiatrist understands what I tell him or her.	local
The psychiatrist answers all of my questions.	local
My psychiatrist is too quiet.	local
Psychiatrists are good about explaining the reasons for tests.	Communication

*Time*:	
Those who provide my psychiatric care sometimes hurry too much when they treat me.	Time spent with doctor
Psychiatrists usually spend plenty of time with me.	Time spent with doctor

*Confidentiality*:	
My psychiatric record is kept safe.	local
I worry about who sees my psychiatric record.	local

*Anxiety*:	
I worry about the future.	local
I worry about my psychiatric care.	local

*Computer Use*:	
The computer gets in the way of the psychiatrist.	local
I am comfortable with the computer in my psychiatrist's office.	local

*In the "Original PSQ-18 subscale" column, "local" indicates the question was based on an unpublished survey that had been drafted by the Principle Investigator during study inception. Otherwise, the question was based on the PSQ-18 and this column shows its PSQ-18 subscale. The Confidentiality, Anxiety, and Computer Use subscales contain locally drafted questions only and are not part of the original PSQ-18 scoring system. PSQ-18 questions belonging to the "Financial Aspects" and "Accessibility & Convenience" subscales were not used.

### Setting & Subjects

Between November 2004 and December 2005, 161 pre-implementation subjects were recruited. A total of 141 Post-implementation surveys were completed Between December 2007 and December 2008. The 24-month interim between collection periods resulted from unanticipated extensions to the EHR implementation date. It also included a four-month acclimation period between full-scale implementation and the beginning of post-implementation recruitment. This acclimation period was intended to prevent the capture of transient results as physicians became more proficient with using the EHR.

All subjects were adult, ambulatory outpatients seen in the University of New Mexico Psychiatric Center (UNM-PC) Continuing Care Clinics. Approximately 2000 chronically mentally ill patients attend these clinics, which are staffed by approximately 10 attending physicians, 5 residents, two certified nurse practitioners, and 10 nurses. Approximately 20 to 40 patients per day are treated for a wide range of psychiatric disorders, including mood, psychotic, anxiety, and personality disorders. Treatment focuses on medication management, although short term psychotherapies are used for select patients. Although case management is widely employed, the vast majority of patients are stabilized on medication and live independently in the community. Dually-diagnosed patients do attend these clinics, but most patients whose primary diagnosis is substance use-related are seen at a different UMN facility. Additionally, patients with dementia or developmental disorders attend other clinics and were therefore not sampled. Those that spoke no English (estimated to be less than 1% of the clinic population) were excluded from the study due to limited bilingual resources. Patients who required psychiatric hospital admission directly from their clinic appointment were excluded from the sample population. During the study period there were no significant changes to the clinic routine other than EHR implementation.

### Consent & Procedure

Potential subjects were approached as they checked out from their outpatient appointments and asked if they would like to participate in a research project investigating the effect of computer use on the patient-psychiatrist relationship. Using a protocol based on order of arrival at the checkout desk, we attempted to approach every patient who checked out from clinic during the data collection periods. If the subject indicated interest, they were taken to an office or secluded area of the waiting room where the purpose, risks, and voluntary nature of the study were fully explained to them. Those that continued to express an interest in participating gave written consent. Each subject was permitted to complete only one satisfaction survey in each study period.

We obtained the participants' written consent for a psychiatric record review and manually recorded their most recent primary diagnosis from their psychiatric record. For comparison of the pre- and post-implementation groups, we also collected race, age, and sex from their hospital record.

### Data Analysis

Target enrollment was 160 subjects per group. This would allow unpaired, two-tailed t-tests to detect a 5% change in survey responses with a 5% chance of Type I error, 20% chance of Type II error, and a standard deviation of 0.8 (on a five-point Likert scale). Because actual enrollment was less than our target, the smallest significant effect size became 7% while maintaining the same chance of Type I and Type II error.

The internal consistency reliability of the composite survey was assessed using standardized Cronbach's coefficient alpha. Comparison between pre-implementation and post-implementation groups was by chi-square tests for categorical variables and by two-tailed, unpaired t-tests for continuous variables. All t-tests used pooled variance except for the "Overall" subscale of the Mood stratum which used the Welch approximation to degrees of freedom due to unequal variances. All statistical analyses and graphics were prepared using version 2.9.0 of the open source and freely available R programming language and environment for statistical computing [47].

## Results

### Comparison of Groups

A total of 161 pre-implementation and 141 post-implementation surveys were initially collected. After eliminating redundant surveys, patient withdrawal, or unclear inclusion criteria found on subsequent review, we were left with 149 pre-implementation and 137 post-implementation surveys. During data analysis, infrequently reported races or infrequently given primary diagnoses were combined into "Other" categories. Table 2 compares demographic characteristics of the pre- and post-implementation groups. The pre-implementation and post-implementation groups were similar with respect to age, race, sex, and primary diagnosis. Characteristics of non-responders were not recorded.

**Table 2:** Comparison of groups

	Pre-implementation	Post-implementation	χ^2 ^(t for age)	df	p
**Number of respondents**	149	137			

**Average age (years)**	49.9	47.6	t = 1.823	284	0.07

**% female (n)**	50% (75)	55% (75)	0.747	1	0.39

					

Race*:			2.654	2	0.27

**Caucasian**	91 (61%)	74 (54%)			

**Hispanic**	39 (26%)	48 (35%)			

**Other**	19 (13%)	15 (11%)			

					

Primary diagnosis**:			0.555	2	0.78

**Mood**	83 (55%)	80 (59%)			

**Psychotic**	48 (32%)	43 (31%)			

**Other**	19 (13%)	14 (10%)			

* Racial categories of "Black or African American" (9 pre-implementation; 7 post-implementation), "American Indian or Alaskan Native" (0 pre-implementation; 2 post-implementation); and "Other" (9 pre-implementation; 6 post-implementation) were combined into one "Other" category for statistical analysis.

** Primary diagnosis categories of "Anxiety" (16 pre-implementation; 8 post-implementation), "Substance use" (0 pre-implementation; 3 post-implementation), and "Other" (3 pre-implementation; 3 post-implementation) were combined into one "Other" category for statistical analysis.

### Survey Internal Consistency Reliability

Table 3 shows the internal consistency reliability for each of the subscales of the composite survey. Only one of our subscales (Technical) met the 0.7 level that is usually considered the minimum for desirable reliability. The Communication & Education subscale scored lower at 0.64, although this value is identical to that of the original PSQ-18 Communication subscale[46]. The three locally generated subscales (Confidentiality, Anxiety, and Computer Use) scored the lowest with standardized alphas of 0.24, 0.59, and 0.38 respectively.

**Table 3:** Internal consistency reliability for composite survey subscales

Composite survey subscale	Standardized alpha	Original PSQ-18 subscale	Original PSQ-18 alpha
Overall	0.58	General	0.75

Technical	0.77	Technical quality	0.74

Interpersonal	0.57	Interpersonal manner	0.66

Communication & Education	0.64	Communication	0.64

Time	0.67	Time Spent with Doctor	0.77

Confidentiality	0.24		

Anxiety	0.59		

Computer Use	0.38		

### Electronic Health Record Associations

**Figure 1:**
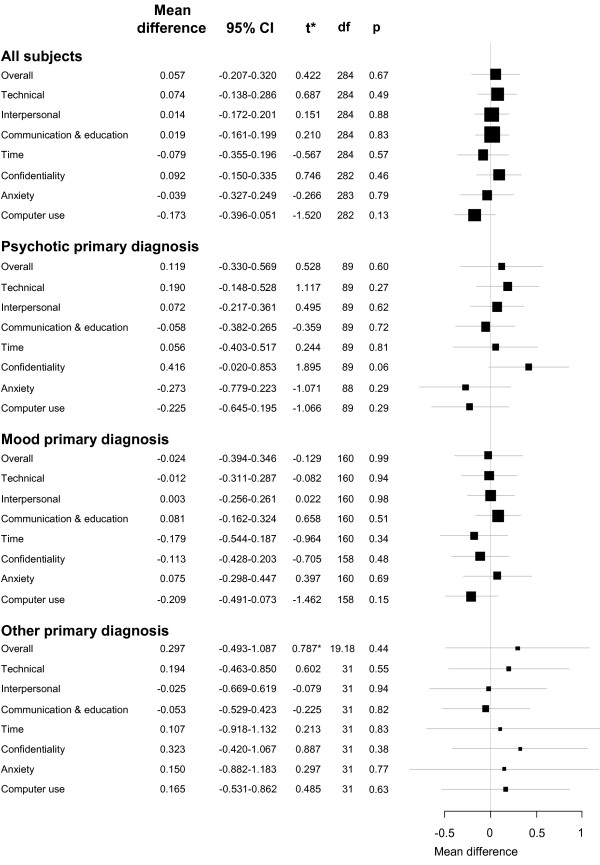
**Change in satisfaction sub-scores**. *All t-tests were based on pooled variance except for the Overall subscale of the Mood stratum which used the Welch approximation to degrees of freedom due to unequal variance.

Figure 1 shows the change in average survey sub-scores before and after EHR implementation. For all subjects, and for subjects stratified by their primary diagnosis, none of the changes reached statistical significance. A post-hoc analysis of average responses for each question separately (rather than grouped into subscales) also showed no significant changes between pre- and post-implementation groups. Raw, mean survey scores are available from the primary author on request.

## Discussion

Although the adoption of Electronic Health Records in the United States has proceeded cautiously, in today's technologically-dependent environment the trend is not likely to be reversed. Instead, emphasis may best be placed on the design of efficient EHR systems [48], determination of best practices for their use [49], attention to communication skills (regardless of the charting modality) [50], and more rigorous collection of data to assess the true impact of EHR use on quality of care, costs, efficiency, and patient views [24].

This study is the first we are aware of that attempted to assess the impact of EHR use on the quality of the patient-psychiatrist relationship in a behavioral health venue. Consistent with several decades of research in the non-psychiatric realm, we found no change in satisfaction scores among adult, psychiatric patients when an EHR was used during outpatient encounters instead of paper charting. Our results should lessen the concerns of behavioral health providers and clinic managers who are hesitant to adopt EHRs because of concerns over potentially negative reactions from their patients. Contrary to our hypotheses and some prior studies, we found no change in patient satisfaction in the Communication & Education, Confidentiality, Anxiety, or any other satisfaction subscales.

Because our samples were powered for a 7% change in satisfaction, Type II error is not likely to explain the lack of significance. Instead, the lack of findings may represent a truly negligible impact of EHR use on the patient-psychiatrist relationship, or it may be due to study limitations.

### Limitations

Interpretation of our results should be tempered in light of its limitations. First, all of our measures were surrogate estimates. We did not attempt to directly measure actual changes in communication patterns, anxiety, or changes in behavior (either on the part of the patient or the psychiatrist). We also did not measure changes in actual patient outcomes.

Second, our survey was not validated. Though it was based on a valid instrument, the changes we made to it resulted in substantially lower internal consistency reliability than the PSQ-18. As well, the PSQ-18 was initially validated in a population that was not exclusively psychiatric and its native validity might not apply as well to the psychiatric population. The ad-hoc analysis, in which the pre- and post-implementation responses to individual questions (as opposed to subscales) were compared, was performed to address this deficiency. Although there is uncertainty in the exact quality being measured by each question, we do know that there were no statistically significant changes to the subjects' ratings of each question. We retained the concept of subscales in our reporting for their face validity and as a way of summarizing data. In order to avoid invalid comparison with the original PSQ-18 subscales, the labels given to our composite subscales were slightly altered from those of the PSQ-18.

The characteristics of any particular EHR system, or the way individual providers use the EHR, can clearly affect patient-physician interaction [51]. We intentionally did not control for the EHR usage patterns of individual providers in order to enhance the sense of patient-provider privacy and to keep the research project strictly separate from any expectations regarding EHR use. Instead, we relied on a large sample size and very low provider turnover to enhance the probability that each provider would be equally represented in the pre-implementation and post-implementation groups.

Fourth, consistent with much survey research of a voluntary nature, our sampling strategy may have biased our samples towards subjects who were more likely to participate in the project because of high satisfaction.

Finally, our use of primary diagnosis offers only a coarse description of the patient pathology and types of personality characteristics that could affect a patient's reactions to EHR use. Many psychiatric diagnoses are co-morbid, particularly mood, personality, and anxiety disorders, and the disorder considered primary on any particular visit may not remain constant. This may have increased the heterogeneity of patient characteristics within each diagnosis strata, while also increasing the homogeneity between strata. Similarly, we did not differentiate between patients with and without personality disorders. Because Axis II disorders are rarely used as the primary diagnoses, we did not attempt to stratify by Axis II pathology. Also, in order to maintain sufficient numbers of subjects in each diagnostic stratum, we grouped diagnoses by major diagnostic category (e.g. "mood disorder") rather than actual primary diagnosis (e.g., "Major Depressive Disorder, recurrent, severe, without psychotic features"). This resulted in only three broad diagnostic strata of "Mood," "Psychotic," and "Other" disorders.

## Conclusion

Consistent with previously published studies on EHR use and patient satisfaction, this study suggests that the use of an Electronic Health Record does not change the overall quality of the patient-psychiatrist relationship.

Patient satisfaction has been shown to affect patient compliance [52,53], treatment outcomes [54,55], malpractice suits [56,57], and the ability to remember instructions [58,59]. Communication skills have consistently shown to affect patient satisfaction [60-62]. Therefore, factors which change communication patterns might also be expected to affect patient outcomes. Psychiatrists and psychiatric patients, who are especially reliant on and sensitive to communication skills, are understandably concerned about the potential impact of EHR use on quality of care provided. This study increases the confidence with which we can extend prior EHR satisfaction studies into the psychiatric realm. While other barriers to EHR adoption do exist, concerns about excessive disruption to the patient-psychiatrist relationship need not be one of them.

## Competing interests

None of the authors report any conflicts of interest, competing interests, or financial disclosures. The National Library of Medicine sponsored this study as part of an Individual Biomedical Informatics Fellowship Grant. The sponsor approved the study design as appropriate for the educational goals of the primary author's (RFS) fellowship, but played no role in the conduct of the study, data collection, data analysis, data interpretation, or preparation of the manuscript.

## Authors' contributions

The primary author (RFS) is responsible for the study concept, initial design, data collection, data analysis, and initial manuscript preparation. MS contributed to study design aspects involving human research, statistical analysis, and data collection methods. RB and PJK participated in psychiatric and biomedical informatics aspects of the study design respectively. All authors read and approved the final manuscript.

## Authors' Information

RFS is an assistant professor in the Department of Biomedical Informatics Research, Training and Scholarship. MS is a professor of Internal Medicine and the Associate Program Director of the UNM General Clinical Research Center Scholars' Program. RB is a Professor of Psychiatry and the Associate Dean for Clinical Affairs of the UNM School of Medicine. PJK is an Assistant Professor and the Director of Biomedical Informatics Research, Training and Scholarship in the UNM Health Sciences Library & Informatics Center. PJK also has an appointment in the Department of Internal Medicine.

## Pre-publication history

The pre-publication history for this paper can be accessed here:

http://www.biomedcentral.com/1471-244X/10/3/prepub
